# Supplementary material for: A Global Screen for Assembly State Changes of the Mitotic Proteome by SEC-SWATH-MS
Source: Cell Syst. 2020 Feb 26;10(2):133–155.e6. doi: 10.1016/j.cels.2020.01.001 (PMC7042714; doi:10.1016/j.cels.2020.01.001)

P51948 | MAT1_HUMAN | MNAT1 CAP35 MAT1 RNF66
Monomer MW [kDa]: 35.823 Monomer expected elution fraction: 49

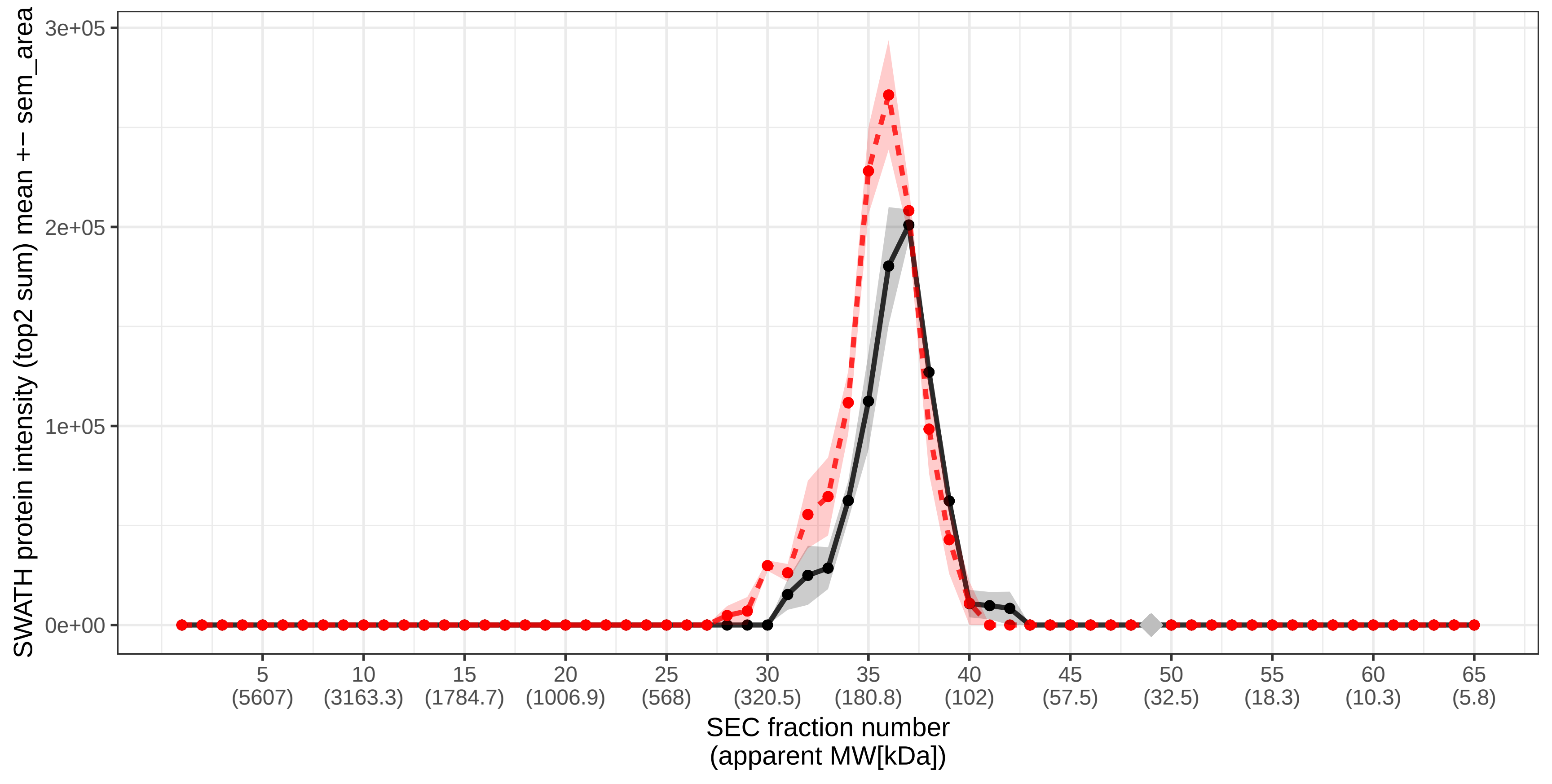

Supplement: Data S1. SEC-SWATH-MS Protein Chromatograms, Related to Figure 1 [file mmc6.zip › SECchrom_P51948_MAT1_HUMAN_MNAT1_CAP35_MAT1_RNF66.pdf]
